# Interpretation of H_2_-TPR from Cu-CHA Using First-Principles Calculations

**DOI:** 10.1021/acs.jpcc.3c07998

**Published:** 2024-03-08

**Authors:** Joachim D. Bjerregaard, Joonsoo Han, Derek Creaser, Louise Olsson, Henrik Grönbeck

**Affiliations:** † Department of Physics and Competence Centre for Catalysis, Chalmers University of Technology, SE-412 96 Göteborg, Sweden; ‡ Chemical Engineering and Competence Centre for Catalysis, Chalmers University of Technology, SE 412 96 Göteborg, Sweden

## Abstract

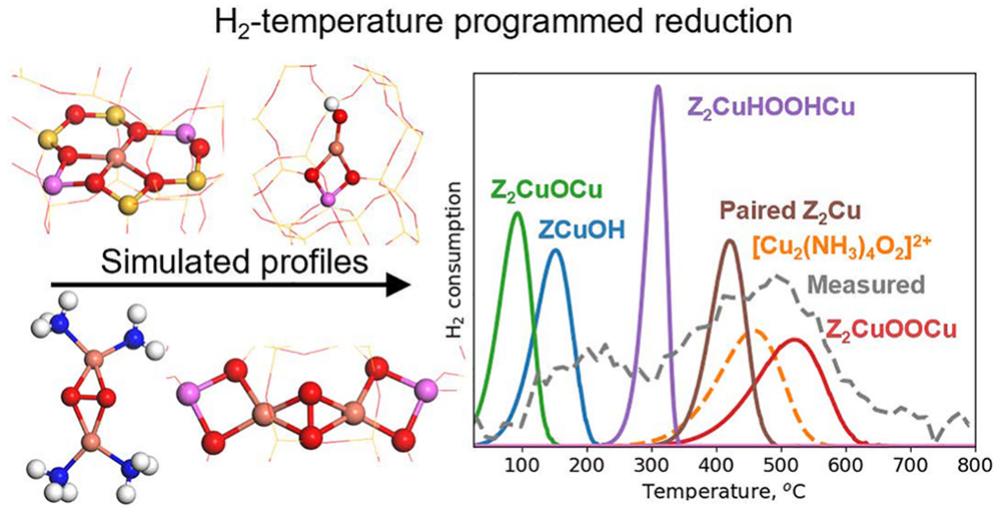

Temperature-programmed reduction and oxidation are used to obtain information on the presence and abundance of different species in complex catalytic materials. The interpretation of the temperature-programmed reaction profiles is, however, often challenging. One example is H_2_ temperature-programmed reduction (H_2_-TPR) of Cu-chabazite (Cu-CHA), which is a material used for ammonia assisted selective catalytic reduction of NO_x_ (NH_3_-SCR). The TPR profiles of Cu-CHA consist generally of three main peaks. A peak at 220 °C is commonly assigned to ZCuOH, whereas peaks at 360 and 500 °C generally are assigned to Z_2_Cu, where Z represents an Al site. Here, we analyze H_2_-TPR over Cu-CHA by density functional theory calculations, microkinetic modeling, and TPR measurements of samples pretreated to have a dominant Cu species. We find that H_2_ can react with Cu ions in oxidation state +2, whereas adsorption on Cu ions in +1 is endothermic. Kinetic modeling of the TPR profiles suggests that the 220 °C peak can be assigned to Z_2_CuOCu and ZCuOH, whereas the peaks at higher temperatures can be assigned to paired Z_2_Cu and Z_2_CuHOOHCu species (360 °C) or paired Z_2_Cu and Z_2_CuOOCu (500 °C). The results are in good agreement with the experiments and facilitate the interpretation of future TPR experiments.

## Introduction

Temperature-programmed reduction with H_2_ (H_2_-TPR) is widely used in heterogeneous catalysis to characterize materials.^1^ The method is straightforward and works by flowing H_2_ over a catalyst, while gradually increasing the temperature. By measuring the consumption of H_2_ during the temperature ramp, information on the chemistry and reducibility of the catalyst material is obtained. The TPR profiles are, however, often complex with many overlapping features, which makes the interpretation ambiguous.

H_2_-TPR has been extensively used to characterize copper-functionalized zeolites to describe and quantify different Cu species.^2−12^ The copper-exchanged small pore zeolite chabazite (Cu-CHA) is used for several important applications. Cu-CHA is presently a state-of-the-art catalyst for deNOx by ammonia-assisted selective catalytic reduction (NH_3_–SCR) in diesel-aftertreatment systems.^13−16^ Furthermore, Cu-CHA has been explored for one-step conversion of methane to methanol.^17−19^

The assignment of Cu species from H_2_-TPR in Cu-CHA is challenging as H_2_ consumption depends on the detailed composition of the catalyst (Si/Al ratio and Cu loading), synthesis method, the oxidation state of Cu (Cu^I^ or Cu^II^), location of Cu ions (e.g., 6- or 8-membered rings), and ligands bound to Cu.^2−5,20^ An additional complication is the dynamic character of the Cu species, which is known to depend critically on the pretreatment of the catalyst. For example, exposure of the catalyst to NH_3_ at low temperature results in the formation of mobile [Cu(NH_3_)_*x*_]^+^ (*x* ≥ 2) complexes,^21−24^ and O_2_ adsorption on the NH_3_-solvated complexes yields peroxo complexes [Cu_2_(NH_3_)_4_O_2_]^2+^. [Cu(NH_3_)_2_]^+^ and [Cu_2_(NH_3_)_4_O_2_]^2+^ have been suggested to be important for the low-temperature NH_3_-SCR reaction.^25−27^ The NH_3_ ligands desorb at high temperatures yielding framework-bound Cu species where ZCuOH and Z_2_Cu are two examples.^28^ Here Z denotes the Al environment with Z being a one-Al environment and Z_2_ being a two-Al environment.

H_2_-TPR peaks for Cu-CHA are commonly observed at ∼220, ∼360, and ∼500 °C;^2−4,6,10−12^ however, the assignments of these peaks are conflicting. The low-temperature peak at 220 °C is often assigned to reduction of ZCuOH species;^3,6,10^ however, peaks at similar temperatures have also been assigned to the reduction of CuO clusters.^5,12^ The assignments of the two peaks at 360 and 500 °C are more challenging, and the peaks are not always present in the TPR profile.^2,3,6^ The peak at 360 °C has been suggested to be the reduction of either ZCuOH or Z_2_Cu.^6,10,12^ The peak at 500 °C is typically assigned to Z_2_Cu.^10,12^ Moreover, it has also been suggested that both the 360 and the 500 °C peaks are related to Z_2_Cu with different atomic structures^10^ or, alternatively, pristine or hydrated Z_2_Cu.^6^ H_2_-TPR peaks are sometimes observed also at very high temperatures (>800 °C) and are in this case related to the formation of metallic Cu.^3,4^ It should be noted that the comparison of the H_2_-TPR profiles between different studies is difficult as the H_2_ consumption depends on the Si/Al ratio (SAR), Cu loading, and sample pretreatment.^2,3,6^ Because of the dynamic character of the Cu species, the actual Cu species present in the investigated samples depends sensitively on the history of the samples.

The interpretation of the H_2_-TPR profiles is important as it often is used to develop atomic-level understanding of the catalytic reaction. For example, conclusions regarding the H_2_-TPR profiles have been used to quantify the ratio between ZCuOH and Z_2_Cu species to understand trends in hydrothermal stability^29^ and sulfur poisoning.^6^ The H_2_-TPR determined ratio between ZCuOH and Z_2_Cu has, moreover, been used as input data for reactor models of Cu-CHA.^30^

Although H_2_-TPR has been used extensively on Cu-CHA, there is an ambiguity as to which Cu species that are responsible for the H_2_ consumption, and the use of the technique to make structure–function links is currently hampered by the lack of chemical understanding of the active sites for H_2_-TPR.

Herein we use density functional theory (DFT) and microkinetic modeling in combination with TPR measurements to study the reaction of H_2_ with different Cu species. The DFT calculations are performed for a range of Cu species known to be present in Cu-CHA. The DFT results are used to construct a microkinetic model to simulate the H_2_-TPR profiles. We study both NH_3_-solvated Cu species, which are relevant for low-temperature NH_3_-SCR and framework-bound species, which are present during high-temperature operation. The experiments are performed by preparing solvated and framework bound Cu species as well as Cu species formed during SCR conditions. The study shows that combining DFT-based microkinetic modeling and H_2_-TPR measurements offers detailed knowledge of the Cu-CHA material, which enables a link between structural properties and catalyst function.

## Theoretical and Experimental Methods

### DFT Calculations

Spin-polarized density functional theory calculations are performed with the Vienna Ab initio Simulation Package (VASP),^31,32^ version 5.4.4. The Kohn–Sham orbitals are expanded with plane waves using a cutoff value of 480 eV. The interaction between the valence electrons and the core is described with the plane augmented wave (PAW) method.^33,34^ The valence electrons considered for each atom are Cu(11), Si(4), Al(3), O(6), N(5), and H(1). The exchange-correlation term is approximated using the Perdew–Burke–Ernzerhof (PBE) functional.^35^ The PBE functional is augmented with a Hubbard-*U* term for Cu 3d to describe the localization of these orbitals,^36^ and a correction^37^ is added to describe the weak van der Waals forces for species in the zeolite. The converging criteria for the SCF loop is set to 10^–5^ eV. The structures are considered to be at a minimum if the norm of all forces acting on the atoms is less than 0.02 eV/Å. Climbing image nudged elastic band (CI-NEB)^38,39^ is used to locate the transition states, which are confirmed by vibrational analyses. The vibrational analysis is performed using the finite difference method. The reaction landscapes are reported with zero-point corrections. Bader charge analyses are performed using the implementation by Henkelmann and co-workers.^40,41^ To explore the flat energy landscape, ab initio molecular dynamic (AIMD) simulations are performed and structures along the trajectory are relaxed. The temperature is set to 300 K and is controlled by a Nosé–Hoover thermostat^42,43^ in the NVT ensemble. A hexagonal unit cell consisting of 36 Si and 72 O atoms is used to model the CHA zeolite. The system is treated with a Si/Al ratio between 5 and 35. The Al site is charged compensated by either H or Cu.

### Simulation of H_2_-TPR

We construct a microkinetic model to simulate the kinetics of the studied H_2_ reactions during a temperature ramp. In the microkinetic model, a set of coupled differential equations are solved.
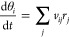
1θ_*i*_ is the fractional coverage of species *i*, *v*_*ij*_ is the stoichiometry coefficient for species *i* and reaction *j*, and *r*_*j*_ is the rate of reaction *j*. The coupled differential equations are solved numerically in Python using the solve_ivp command from the scipy.integrate package, with the implicit multistep variable-order (BDF) method. In temperature-programmed desorption (TPD), the time-dependent rate of desorption for species *i* (*r*_des_) is described by^44^
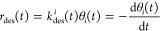
2The rate of desorption *r*_des_ depends on the rate constant *k*_des_^*i*^ and the fractional coverage of the adsorbed species θ_*i*_. The temperature of the system is a function of time (*t*):
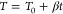
3Here, *T*_0_ is the starting temperature and β is the heating rate. Equation 2 can be adopted to TPR measurements,^45^ where instead the consumption (adsorption) of H_2_ is monitored:
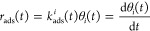
4For TPR, the rate constant of the adsorption (*k*_ads_^*i*^(*t*)) is used instead of *r*_des_. The rate constants are computed from transition state theory (TST).
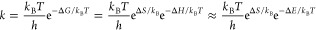
5*k*_B_ is the Boltzmann constant, *T* is temperature, *h* is Planck’s constant, and Δ*G* is the change in the Gibbs free energy between the transition state and the initial state. The enthalpy is approximated as the zero point corrected DFT energy, *E*. For adsorbed H_2_, the entropy is evaluated via the vibrational partition function, and for gas phase H_2_, the entropy is evaluated via the partition function for vibration, translation, and rotation. For the dissociative adsorption of H_2_, the rate constant is described by collision theory.
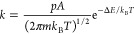
6*p* is the partial pressure, *A* is the area, which is set to the pore size of the zeolite corresponding to the eight-membered ring, *m* is the mass, and Δ*E* is the change in zero-point corrected DFT energy between the transition state and initial state. To ensure thermodynamic consistency, the desorption rate, *k*_des_, is calculated from the equilibrium constant, *K*.
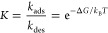
7The desorption of H_2_O is modeled as an irreversible reaction with the following rate constant.
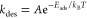
8*E*_ads_ is the zero-point corrected desorption energy, and the prefactor for the desorption is set to 10^12^ s^–1^.

### Cu-CHA Synthesis

A Cu-CHA powder is prepared by first synthesizing a Na form of CHA (Na-CHA) using a direct hydrothermal synthesis method. In a second step, an NH_4_ form of CHA (NH_4_-CHA) is prepared using an ion-exchange method with ammonium nitrate (NH_4_NO_3_). A detailed account of the synthesis procedure is reported elsewhere.^2,12,46^ The NH_4_-CHA powder is used to prepare the Cu^II^-exchanged CHA (Cu-CHA) powder using the incipient wetness impregnation method. 0.160 g of copper nitrate (Cu(NO_3_)_2_·2.5H_2_O, Sigma-Aldrich, pentahydrate) is dissolved into 3 g of ethanol as a mixture. 2 g of the prepared NH_4_-CHA powder is introduced into the copper-containing mixture, and the resulting mixture is continuously stirred for 15 min. Subsequently, the mixture is dried at room temperature overnight and thereafter well-crushed to a fine powder form. The crushed Cu-CHA powder is calcined at 600 °C for 8 h, followed by 750 °C for 2 h at a heating rate of 2 °C/min. Elemental composition of the Cu-CHA powder is analyzed through an inductively coupled plasma mass spectrometry (ICP) analysis for Si, Al, and Cu. The ICP results show an Si/Al ratio = 11.4 and 2 wt % Cu.

### Temperature-Programmed Reduction with H_2_

Temperature-programmed reduction with H_2_ (H_2_-TPR) is performed with the prepared Cu-CHA sample. Prior to the test, the sample is exposed to standard SCR conditions (400 ppm of NH_3_/NO, 5% H_2_O, 10% O_2_, Ar Bal.) at 750 °C for 5 h to degreen the Cu-CHA powder. Thereafter, the resulting Cu-CHA powder is sieved to obtain a narrower particle size range (180–250 μm). The resulting Cu-CHA sample is loaded inside a vertical quartz tube within a differential scanning calorimeter (Sensys DSC calorimeter, Setaram).

**Figure 1:**
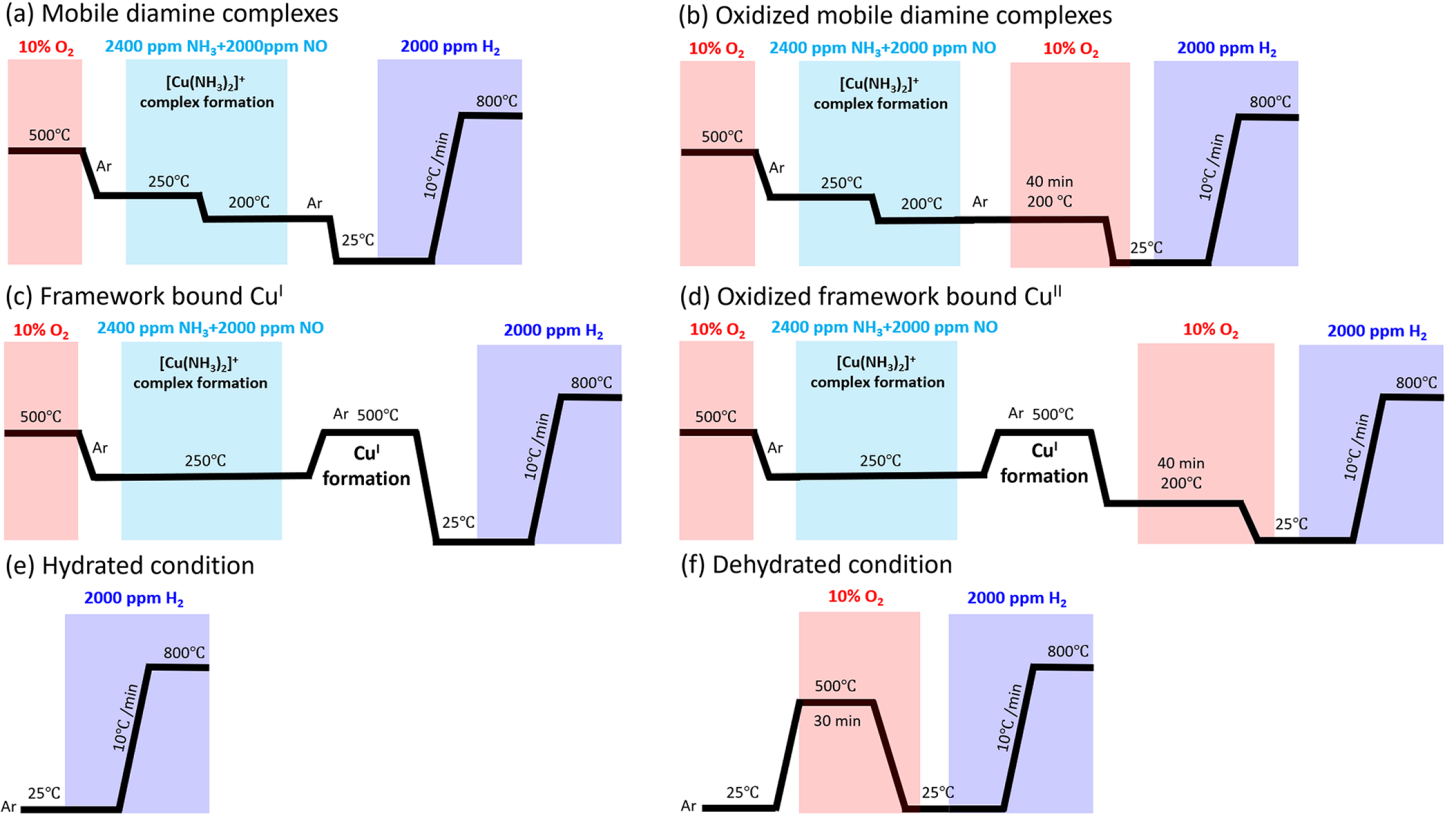
H_2_-TPR test protocols for four different copper states and comparison of the hydrated and dehydrated conditions over the Cu-CHA. The TPR experiments examines (a) mobile diamine complex, (b) oxidized mobile diamine complex, (c) framework bound Cu^I^, (d) oxidized framework bound Cu^II^, (e) hydrated condition, and (f) dehydrated condition. Total flow rate: 20 N mL/min; heating rate: 10 °C/min.

The samples are pretreated to obtain six dominant types of copper species following the H_2_-TPR test protocols in Figure 1.

**Figure 2:**
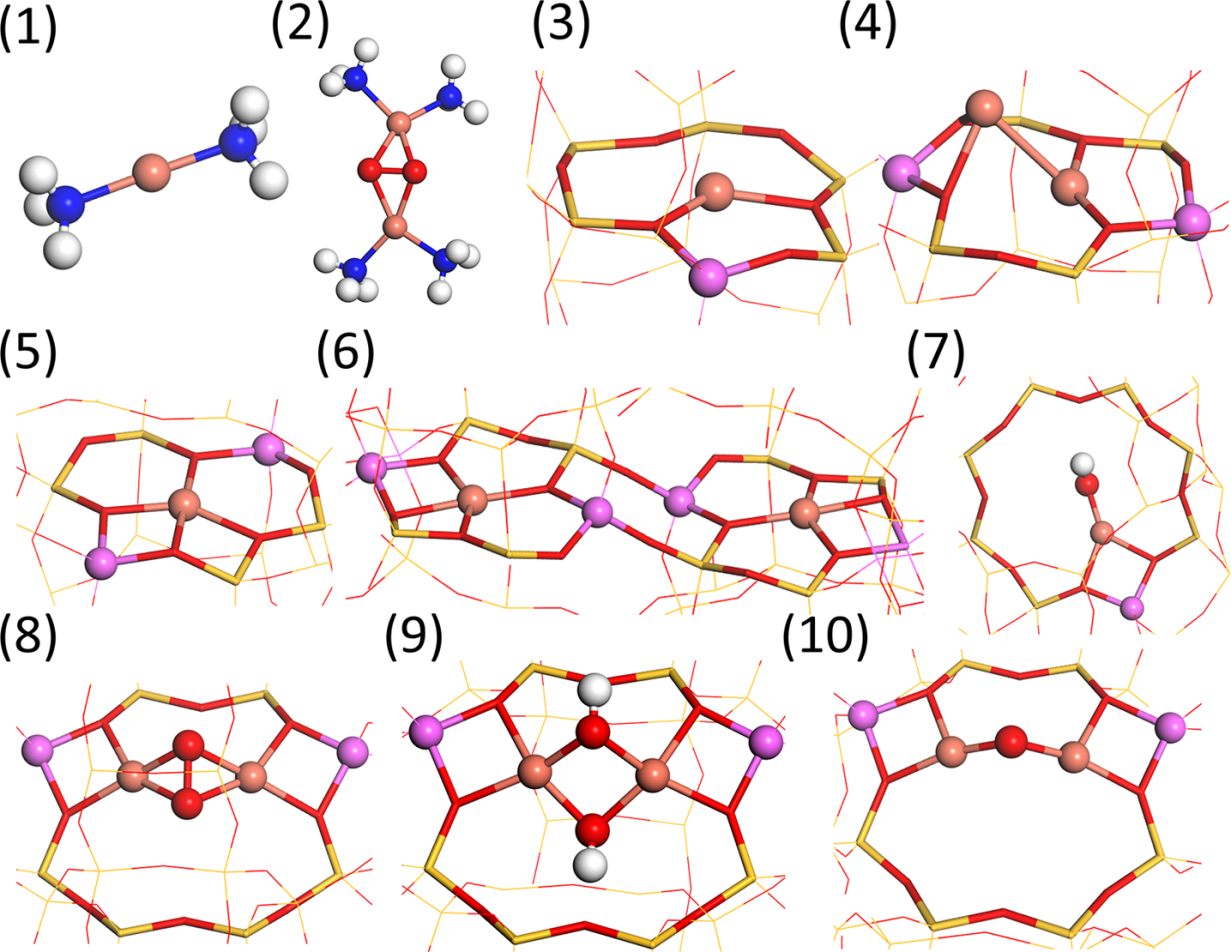
Considered Cu species: (1) [Cu(NH_3_)_2_]^+^, (2) [Cu_2_(NH_3_)_4_O_2_]^2+^, (3) ZCu, (4) Z_2_Cu_2_, (5) Z_2_Cu, (6) paired Z_2_Cu, (7) ZCuOH, (8) Z_2_CuOOCu, (9) Z_2_CuHOOHCu, and (10) Z_2_CuOCu. For configurations not bonded to the framework (a and b), the cage is not shown for clarity. Atomic color codes: H (white), N (blue), O (red), S (yellow), and Cu (bronze).

Initially, five of the samples were pretreated at 500 °C under oxidizing conditions (10% O_2_) for 30 min, which would remove most of the water for the samples as well as oxidize the copper. In the first experiment the mobile diamine complex is targeted (Figure 1a). This is done by exposing the Cu-CHA sample to 2400 ppm of NH_3_ + 2000 ppm of NO through reduction half-cycle according to the standard SCR mechanism at low temperature.^47,48^ Thereafter, the oxidized mobile diamine complex is examined (Figure 1b) by exposing the mobile diamine complex to O_2_ at 200 °C for 40 min. The diamine complex efficiently activates O_2_ molecule at 200 °C, leading to the oxidized mobile diamine complex (Figure 2b).^47^ The method shown in Figure 1c focuses on the framework bound Cu^I^ and the oxidized framework bound Cu^II^ (Figure 1d) by treating the mobile diamine complex to high temperature at 500 °C for 1 h. Under the thermal treatment, NH_3_ desorbs from the complex leading to the framework bounded Cu^I^.^47^ The resulting framework bound Cu^I^ activates the O_2_ molecule at 200 °C, leading to the framework bound Cu^II^.^47^Figures 1c and 1d therefore lead to framework bound Cu^I^ and Cu^II^, respectively. The degreened Cu-CHA powder is exposed to air for several days to allow for water uptake to mimic hydrated conditions as shown in Figure 1e to identify ZCuOH at low temperature by comparing dry conditions (Figure 1f). The mobile diamine complexes, oxidized mobile diamine complexes, and framework-bound Cu^I^ species have previously been identified using X-ray absorption spectroscopy on Cu-CHA samples exposed to similar pretreatments as in the present study.^21,22,49,50^

After each pretreatment, the temperature is cooled to room temperature. The H_2_-TPR is started by exposing the sample to 2000 ppm of H_2_ at RT for 30 min and a temperature ramp where the temperature is increased to 800 °C at heating rate of 10 °C/min. The temperature is thereafter kept at 800 °C for 30 min. Effluent gases are monitored with a mass spectrometer (Hiden HPR-20 QUI MS) for H_2_ (*m*/*e* = 2), NH_3_ (15), H_2_O (18), Ar (20), N_2_ (28), NO (30), O_2_ (32), N_2_O (44), and NO_2_ (46). Ar is used as a balance gas, and the total flow rate was maintained at 20 N mL/min during the entire measurements.

## Results

### Considered Cu Species

The state of the Cu ions in Cu-CHA depends sensitively on the reaction conditions.^21^ Here we consider the structures shown in Figure 2. At low-temperature NH_3_-SCR conditions, the Cu ions are solvated, forming mobile [Cu^I^(NH_3_)_2_]^+^ complexes (structure (1)).^21−24^ A pair of mobile complexes can react with O_2_, forming peroxo complexes [Cu_2_^II^(NH_3_)_4_O_2_]^2+^ (structure (2)). Structures (1) and (2) are important intermediates in the low-temperature NH_3_-SCR reaction.^25−27^ At higher temperatures, NH_3_ desorbs, and the Cu ions bind to the zeolite framework, forming, for example, structures (3) and (4). (3) is a single Cu^I^ ion in the six-membered ring, whereas (4) is paired Cu^I^ ions balanced by two Al sites. The Cu configurations Z_2_Cu^II^ (structures (5) and (6)) and ZCu^II^OH (structure (7)) are structures frequently discussed in the literature.^2,3,6^ (5) is an isolated Cu^II^ ion located in a six-membered ring, and (6) are paired Cu^II^ ions located in six-membered rings. The ZCu^II^OH species in structure (7) is located in the eight-membered ring. If the catalyst is exposed to O_2_ at high temperature, a pair of Cu ions may adsorb an oxygen molecule, forming a framework bound peroxo species Z_2_Cu^II^OOCu^II^ (structure (8)) in an eight-membered ring.^47^ Structure (8) has been suggested to be important for the methane-to-methanol reaction.^17,18^ Structure (9) (Z_2_Cu^II^HOOHCu^II^) and structure (10) (Z_2_Cu^II^OCu^II^) are two additional structures in the eight-membered ring that have been proposed to be present in Cu-CHA.^17−19^ (9) could potentially be formed by the reaction of two ZCu^II^OH complexes.

### H_2_ Adsorption over Cu Species in Cu-CHA

**Figure 3:**
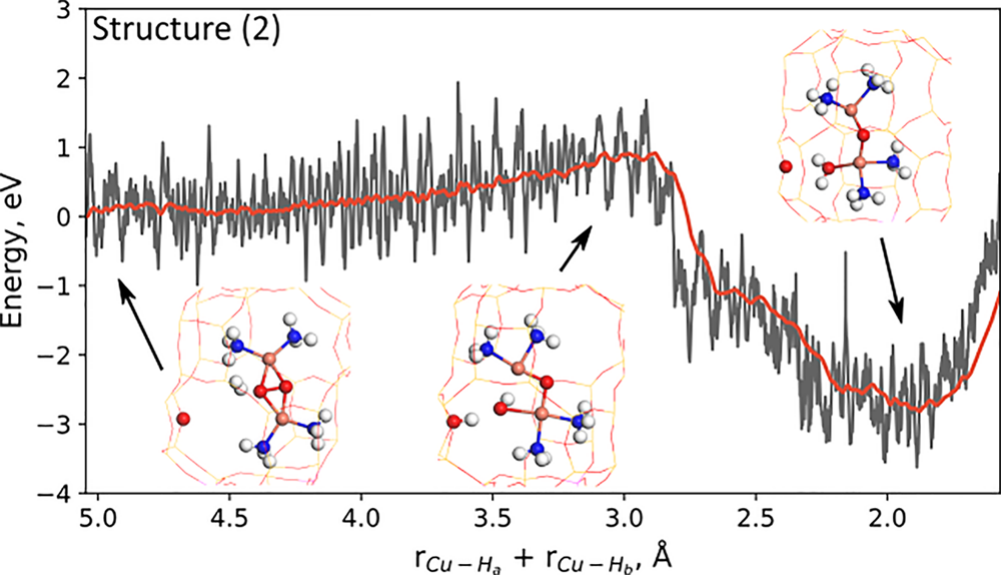
Constrained AIMD simulations of the energy profile for the reaction of H_2_ with the mobile peroxo dimer [Cu_2_(NH_3_)_4_O_2_]^2+^. The gray line is the energy, and the red line is the rolling average of the energy. Atomic color codes as in Figure 2.

**Figure 4:**
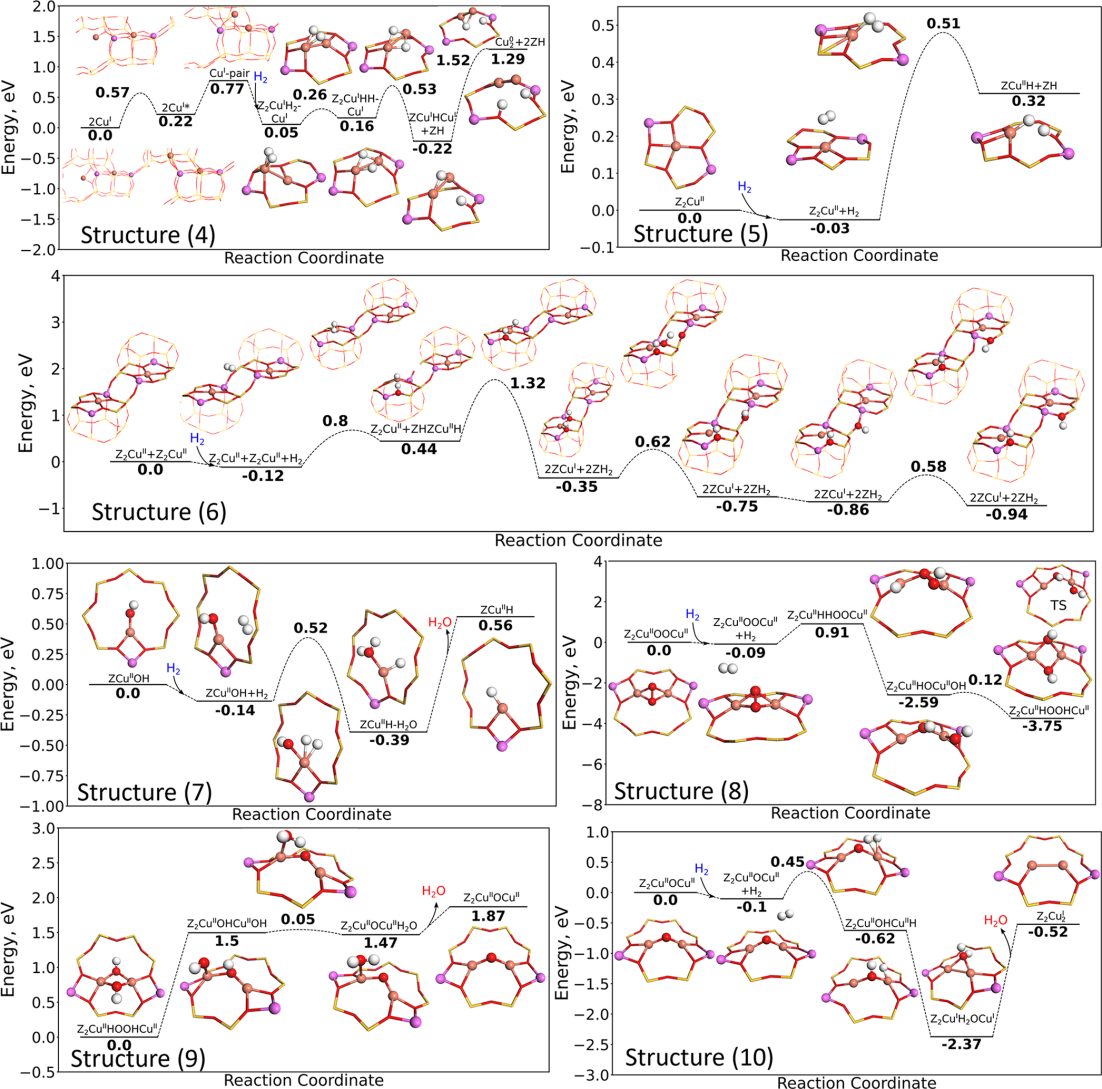
Reaction landscape for the dissociation of H_2_ over (4) Z_2_Cu_2_, (5) Z_2_Cu, (6) paired Z_2_Cu, (7) ZCuOH, (8) Z_2_CuOOCu, (9) Z_2_CuHOOHCu, and (10) Z_2_CuOCu. Atomic color codes as in Figure 2.

The potential energy diagrams for H_2_ adsorption over the different types of Cu species are presented in Figures 3 and 4. We did not find it possible to adsorb or dissociate H_2_ over structure (1) ([Cu(NH_3_)_2_]^+^); thus, we do not expect this complex to consume H_2_ during TPR measurements.

The peroxo complex [Cu_2_(NH_3_)_4_O_2_]^2+^ (structure (2)) is flexible, and we were not able to locate a transition state for H_2_ dissociation using CI-NEB. Instead, constrained AIMD simulations were performed to probe the reaction barrier. The simulation is shown in Figure 3. The collective variable is the sum of the bond lengths between the hydrogen atoms and the Cu (see the Supporting Information for more information). The energy barrier for H_2_ adsorption is 0.92 eV. H_2_ dissociates over a Z site before forming H_2_O in the final state. The Cu ions are in a +2 oxidation state in both the initial and final states, and the two oxygen atoms are reduced (O^1–^ ⇒ O^2–^). Reacting a second H_2_ molecule with the remaining oxygen atom leads to a pair of [Cu(H_2_O)(NH_3_)_2_]^+^ complexes with Cu in the oxidation state +1 (see the Supporting Information).

The potential energy diagrams for H_2_ adsorption and dissociation over the Cu species are collected in Figure 4. Four different Cu species are considered for the framework-bound Cu complexes without extraframework oxygen ligands, namely isolated (structure (3)) and paired ZCu (structure (4)) and isolated Z_2_Cu (structure (5)) and paired Z_2_Cu (structure (6)). The results for isolated ZCu (structure (3)) is shown in the Supporting Information. H_2_ adsorbs weakly on isolated ZCu (−0.28 eV), and the subsequent reactions are highly endothermic.

A pair of ZCu (structure (4)) can dissociate H_2_, and the reaction landscape for the reaction is shown in Figure 4. To dissociate H_2_, the Cu ions diffuse to form a Cu pair. The starting structure 2Cu corresponds to two ZCu ions located in an eight- and six-membered ring. The first step involves diffusion of the Cu ion in the eight-membered ring to an adjacent eight-membered ring forming structure 2Cu*. The diffusion is endothermic by 0.22 eV and associated with a barrier of 0.57 eV. The next step, which is the formation of the final Cu pair, is endothermic by 0.55 eV with a Cu–Cu distance of 2.5 Å (structure Cu-Pair). The short distance between the two Cu ions is necessary to dissociate H_2_. H_2_ adsorbs on one of the Cu ions with a binding energy of −0.72 eV forming Z_2_CuH_2_Cu. H_2_ dissociates over the pair with a barrier of 0.26 eV, forming Z_2_CuHHCu. In this structure, the Cu ions remain in a +1 oxidation state, and the hydrogen atoms remain neutral. One of the hydrogen atoms can migrate to a Brønsted site forming Z_2_Cu–H–Cu + ZH, where ZH denotes the Brønsted acid site. The next step is the simultaneous reduction of the Cu ions into a Cu dimer and two Brønsted acid sites (Cu_2_ + 2ZH). This leads to two Brønsted acid sites and a Cu_2_ dimer with a bond length of 2.24 Å. The reduction has a high barrier of 1.52 eV and is endothermic by 1.51 eV. The reaction landscape for H_2_ adsorption and dissociation over two ZCu suggests that this reaction is unfavorable. ZCu have been reported to be reduced at high temperatures^3^ and associated with the destruction of the framework,^4^ a process that we have not considered here.

For isolated Z_2_Cu (structure (5)), the dissociation of H_2_, results in a Cu–H complex and a Brønsted acid site (ZCuH + ZH). The barrier is 0.51 eV, and the reaction is endothermic by 0.35 eV; thus this reaction is not favored. The Cu ion in structure ZCuH + ZH has a magnetic moment of 0.47, which implies that the Cu ion remains in a +2 oxidation state. This is corroborated by a Bader charge analysis, showing that the charge on the Cu ion changes from +1.17*e* to +0.90*e* in the reaction. The hydrogen atom bound to the Cu ion is slightly negatively charged (−0.28*e*), and the hydrogen atom bound to the Brønsted site has a positive charge (+0.59*e*). Thus, the Cu ion remains in the +2 oxidation state because of the formation of a formal H^–^ ion.

An alternative route for the reaction of H_2_ with Z_2_Cu, is the reaction of one H_2_ molecule over paired Z_2_Cu sites (structure (6)), forming 2ZCu + 2ZH. H_2_ dissociates over Cu forming Z_2_Cu + ZHZCuH, which is the same step as for isolated Z_2_Cu. The dissociation of H_2_ is endothermic by 0.56 eV and has a barrier of 0.8 eV. One of the hydrogen atoms can migrate from Cu to a Brønsted site (2ZCu + 2ZH). The two Cu ions are reduced to Cu^I^ upon the hydrogen diffusion. The hydrogen diffusion has a large barrier of 1.32 eV, and the reaction is exothermic by −0.79 eV. In this 2ZCu + 2ZH configuration, 2 Al ions are associated with two Brønsted acid sites and a Cu ion, thus, a local structure that is not fully charge stabilized. Hydrogen diffusion to a nearby Al ion stabilizes the local charge. This second diffusion step has a barrier of 0.62 eV and is exothermic by −0.4 eV. Further diffusion of the hydrogen leads to more stable structure. First, the hydrogen can rotate lowering the energy by −0.11 eV. The last step is hydrogen diffusion to an oxygen in the six-membered ring, lowering the energy by −0.08 eV with a barrier of 0.58 eV.

Turning to the oxygen-containing Cu species, we start with H_2_ reacting with ZCuOH (structure (7)). H_2_ dissociates over the Cu ion forming a Cu–H complex with H_2_O bound to the Cu ion (structure ZCuH–H_2_O). The barrier is 0.52 eV, and the reaction is exothermic by −0.25 eV. The Cu ion in ZCuOH remains in a +2 oxidation state, and the hydrogen bound to Cu is slightly negatively charged (−0.18*e*), which is similar to the Z_2_Cu case. The formed H_2_O has a desorption energy of 0.95 eV forming ZCuH. As the hydrogen atom is negatively charged, it can, in principle, be transferred in an exothermic reaction to another ZCuOH complex forming an H_2_O molecule and two ZCu ions. Thus, paired ZCuOH sites can be reduced by a single hydrogen molecule H_2_ (reaction landscape in the Supporting Information).

An O_2_ molecule can adsorb on a pair of Cu ions forming a framework-bound peroxo species,^47^ Z_2_CuOOCu (structure (8)). H_2_ does not dissociate directly over the oxygen atoms. However, similar to the previous reaction landscapes, H_2_ dissociates over the Cu ions. The dissociation of H_2_ is endothermic by 1.0 eV and results in a Z_2_CuHHOOCu species. Both Cu ions remain in a +2 oxidation state. The hydrogen atom bound to the Cu ion is slightly negatively charged by −0.18*e*, whereas the hydrogen bound to the oxygen atom is positively charged (+0.65*e*). This charge separation is similar to H_2_ over ZCuOH and Z_2_Cu. In a subsequent exothermic step (−3.5 eV), the hydrogen atom bound to the Cu ion migrates to the oxygen (Z_2_CuHOCuOH), reducing the two oxygen atoms (O^1–^ ⇒ O^2–^) The free −OH group can bind to the Cu ion forming a very stable Z_2_CuHOOHCu species (exothermic by −1.16 eV) with a barrier of 0.12 eV.

The formed Z_2_CuHOOHCu species can react further with an additional H_2_ (structure (9)). The Z_2_CuHOOHCu species could also be formed by the reaction of two ZCuOH species (see reaction path in the Supporting Information). H_2_ does not adsorb over Z_2_CuHOOHCu, and the reaction can proceed only after the formation of a Z_2_CuOCuH_2_O species. Z_2_CuOCuH_2_O is formed by breaking a Cu–O bond to form an −OH group bound to a single Cu (Z_2_CuOHCuOH). This reaction step is highly endothermic (1.5 eV). In a next step, the hydrogen atom is transferred to the −OH group in a thermoneutral reaction with a barrier of 0.05 eV. The desorption of H_2_O is associated with a barrier of 0.4 eV forming Z_2_CuOCu. The reaction of Z_2_CuOCu with H_2_ follows the same path for structure (10).

Z_2_CuOCu complexes have also been proposed to exist and formed from the dehydration of Z_2_CuHOOHCu species (structure (10)). H_2_ is in this case dissociated over a Cu ion with a barrier of 0.55 eV forming Z_2_CuOHCuH. The hydrogen atoms can subsequently migrate to the −OH group forming H_2_O, (Z_2_CuH_2_OCu) in an exothermic reaction, whereby the Cu ion is reduced to a +1 oxidation state. The H_2_O molecule is bound to both Cu ions with a Cu–O bond length of 2.00 Å. The desorption energy of H_2_O from this state is 1.85 eV; hence H_2_O is strongly bonded. The DFT calculations suggest that only ions in oxidation state +2 are expected to be reduced to +1 upon H_2_ exposure.

### Simulated H_2_-TPR Profiles

**Figure 5:**
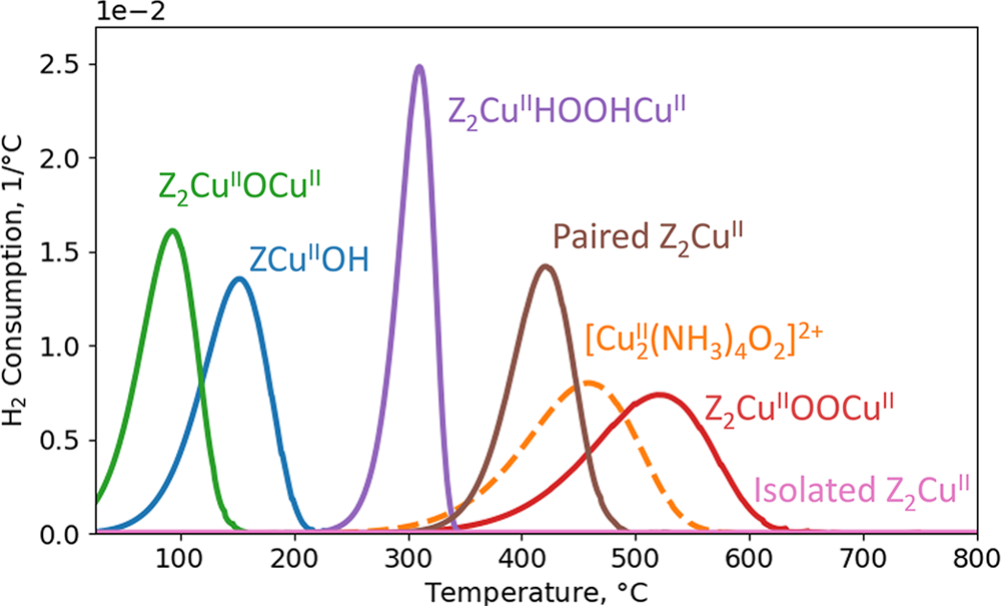
Simulated H_2_ consumption profile during TPR for Z_2_CuOCu, ZCuOH, Z_2_CuHOOHCu, paired Z_2_Cu, [Cu_2_(NH_3_)_4_O_2_]^2+^, Z_2_CuOOCu, and isolated Z_2_Cu. The dashed line indicates that [Cu_2_(NH_3_)_4_O_2_]^2+^ decomposes below the simulated consumption peak. Only one site is considered for each simulation. The heating rate and H_2_ pressure are set to 10 °C/min and 2000 ppm, respectively.

The DFT calculations show that neither the mobile [Cu(NH_3_)_2_]^+^ complex nor the framework-bound ZCu adsorbs H_2_. H_2_ adsorption and dissociation over a pair of ZCu ions are associated with high barriers rendering also this structure improbable for H_2_ consumption at experimental conditions. Thus, these structures are not included in the simulations of the H_2_-TPR profiles. For the remaining species, we simulate H_2_-TPR profiles based on the DFT energy landscapes (Figure 5), treating each Cu species separately. The reactions, including the kinetic parameters, are reported in Table S1.

Z_2_CuOCu consumes H_2_ already at low temperatures with a maximum at 95 °C. ZCuOH has a peak at 150 °C. Z_2_CuHOOHCu consumes H_2_ at 300 °C. Paired Z_2_Cu consumes H_2_ at 420 °C, whereas isolated Z_2_Cu does not consume H_2_ as this reaction is endothermic. [Cu_2_(NH_3_)_4_O_2_]^2+^ consumes H_2_ at 460 °C forming a broad peak. It should be noted that 460 °C is above the temperature for desorption of the NH_3_ ligands. Thus, H_2_ consumption over [Cu_2_(NH_3_)_4_O_2_]^2+^ should experimentally be limited to the low-temperature tail. H_2_ adsorption on Z_2_CuOOCu results in a broad peak at 520 °C.

To summarize, only the Cu species in oxidation state +2 consume H_2_. From the simulated H_2_-TPR profiles, the Cu species can be divided into three groups. The species that consume H_2_ at low temperatures contain a single oxygen atom (Z_2_CuOCu and ZCuOH). Hydrogen can be adsorbed at Z_2_CuHOOHCu and paired Z_2_Cu species at medium temperatures. Species that consume H_2_ at high temperatures contain two oxygen atoms ([Cu_2_(NH_3_)_4_O_2_]^2+^ and Z_2_CuOOCu).

### Measured H_2_-TPR Profiles

**Figure 6:**
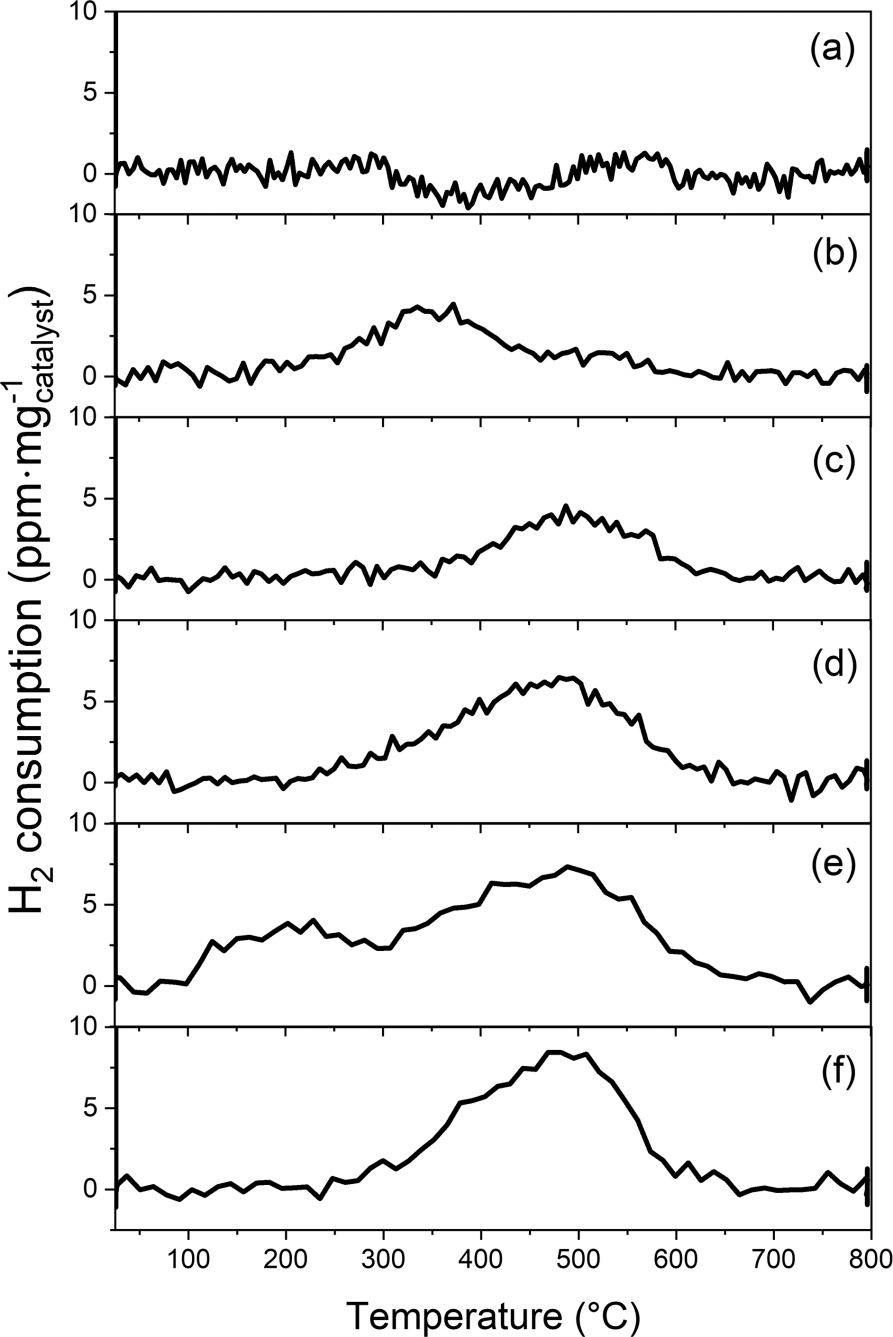
Experimental H_2_ consumption profile during TPR for different pretreatments. The pretreatments for each experiment are shown in Figure 1. The amounts of consumed H_2_ are no consumption, 0.06, 0.06, 0.12, 0.18, and 0.14 μmol/mg_cat_ for panels a, b, c, d, e, and f, respectively.

Results from the H_2_-TPR experiments are reported in Figure 6a–f. The profiles correspond to different pretreatments as shown in Figure 1. Figure 6a shows the H_2_ consumption over the mobile [Cu(NH_3_)_2_]^+^ complexes, which turns out to be negligible. A pair of [Cu(NH_3_)_2_]^+^ can adsorb O_2_, forming [Cu_2_(NH_3_)_4_O_2_]^2+^. The [Cu_2_(NH_3_)_4_O_2_]^2+^ complex consumes H_2_ with a peak at 350 °C (Figure 6b). It is important to note that [Cu(NH_3_)_2_]^+^ and [Cu_2_(NH_3_)_4_O_2_]^2+^ are stable only below ∼300 °C; thus, desorption of NH_3_ is observed during the TPR experiments (see the Supporting Information), indicating that the complexes decompose. For [Cu_2_(NH_3_)_4_O_2_]^2+^, the desorption of NH_3_ occurs simultaneously with the H_2_ consumption. Hence, the amount and temperature at which [Cu_2_(NH_3_)_4_O_2_]^2+^ adsorbs H_2_ cannot be resolved unambiguously. H_2_ consumption over a sample with dominantly ZCu has a peak at 490 °C as shown in Figure 6c. The framework-bound dimer Z_2_CuOOCu (Figure 6d) has a broad peak starting at ∼200 °C with a maximum at 490 °C. The temperature for the main peak is similar to ZCu. The profiles in Figures 6e and 6f are obtained over samples that have been pretreated with SCR conditions (Figure 6e) and SCR conditions followed by a dehydration step (Figure 6f). Figure 6e displays two peaks; a low-temperature peak at 230 °C and a high-temperature peak at 490 °C, possibly with a shoulder at 375 °C. After dehydration (Figure 6e), the low-temperature peak is removed, whereas the higher temperature peak and shoulder are unaffected.

## Discussion

In the literature, the measured H_2_-TPR profiles of Cu-CHA consist generally of three main peaks at 220, 360, and 500 °C.^2−4,6,10−12^ Our measurement for H_2_ consumption over a sample that is pretreated with SCR conditions shows similar low- and high-temperature peaks. The low-temperature peak in the literature is commonly assigned to ZCuOH, whereas the high-temperature peaks are generally assigned to Z_2_Cu.

The combination of a first-principles-based microkinetic model with H_2_-TPR measurements on samples with different pretreatments makes it possible to connect the consumption of H_2_ with specific Cu species in the Cu-CHA samples. In the first-principles-based microkinetic modeling, we have considered a range of different Cu species. At low temperatures, Z_2_CuOCu has a peak in the H_2_ consumption at 95 °C and ZCuOH has a peak at 150 °C. Z_2_CuHOOHCu and paired Z_2_Cu species consume H_2_ at 300 and 420 °C, respectively, and at high temperatures, [Cu_2_(NH_3_)_4_O_2_]^2+^ and Z_2_CuOOCu show H_2_ consumption at around 500 °C. We find that Cu ions in oxidation state +1 do not consume H_2_.

Experimentally, samples prepared with dominantly [Cu(NH_3_)_2_]^+^ do not show any H_2_ consumption, in agreement with the simulations. The sample with [Cu_2_(NH_3_)_4_O_2_]^2+^ shows some H_2_ uptake. However, the peak at ∼350 °C is above the NH_3_ desorption temperature. Thus, the TPR profile should mainly originate from framework-bound Cu species, such as ZCu, ZCuNH_3_, or oxidized framework bound complexes, Cu_*x*_O_*y*_. The simulated profile of [Cu_2_(NH_3_)_4_O_2_]^2+^ shows a peak at 460 °C. However, our simulations do not include the decomposition of [Cu_2_(NH_3_)_4_O_2_]^2+^ above ∼300 °C, and contributions to the H_2_ consumption from [Cu_2_(NH_3_)_4_O_2_]^2+^ should therefore only be expected from the tail at low temperatures.

The measurements of a sample with dominantly ZCu show a peak at 490 °C. This is in disagreement with the DFT calculations and could be related to the presence of paired Z_2_Cu species also in this sample and/or some oxidation. The temperature at which H_2_ is consumed in Figure 6c is similar the temperature of the peak in Figure 6d, where Z_2_CuOOCu was selectively prepared. In the literature,^3,51^ ZCu has been assigned to a peak at very high temperatures (>800 °C). As this is in a temperature region where the CHA structure starts to degrade,^4^ the H_2_ consumption could be connected to the formation of defects in the zeolite framework. Oxidized framework bound Cu shows a peak at 490 °C, which is in fair agreement with the simulated peak at 520 °C.

The sample that has been pretreated in SCR conditions (Figure 6e) shows two peaks at 230 and 490 °C, respectively. The low-temperature peak is close to the simulated peak for H_2_ consumption over ZCuOH, and assigning the low-temperature peak to ZCuOH agrees with previous suggestions.^2,3,6^ Our simulations suggest that the high-temperature peak could be assigned to Z_2_CuOOCu. The simulated profile for the peroxo species [Cu_2_(NH_3_)_4_O_2_]^2+^ has a peak at 460 °C. However, as already discussed, this temperature is higher than the desorption temperature of NH_3_, which means that the [Cu_2_(NH_3_)_4_O_2_]^2+^ species do not contribute directly to the TPR profile. In the literature, the high-temperature peak has previously been assigned to Z_2_Cu. We find that isolated Z_2_Cu does not consume H_2_, whereas paired Z_2_Cu consume H_2_ with a peak at about 420 °C. The computed peak at 420 °C from paired Z_2_Cu agrees also reasonably well with a feature at 360 °C, which is commonly reported in the literature^2,10^ and is typically assigned to Z_2_Cu.

The fact that only paired Z_2_Cu sites consume H_2_, whereas isolated Z_2_Cu does not, suggests that the distribution of Z_2_Cu sites in Cu-CHA plays an important role for the H_2_ consumption. How close the Z_2_Cu species should be to allow for H_2_ consumption cannot be investigated using only one hexagonal unit cell of CHA. However, as both Cu ions are reduced simultaneously, the Cu ions should be close enough to allow for the electron transfer. An increased Cu loading with constant Si/Al ratio should result in a larger fraction of paired Z_2_Cu species. H_2_-TPR experiments on samples with an Si/Al ratio of 9 and different Cu loadings (0.8–3.99%), show an increased H_2_ consumption for the high-temperature peak with higher Cu loading,^10^ which is consistent with our study.

There are alternative distributions of the Al ions for the Cu complexes reported in Figure 2. By changing the location of the Al ions, the reactivity toward H_2_ may change, resulting in a shift of the H_2_-consumption peak. To test the sensitivity, the H_2_ dissociation is calculated for paired Z_2_Cu and Z_2_CuOOCu with different Al distributions. For Z_2_Cu, the Al ions are placed in the six-membered ring, separated by one Si atom, and for Z_2_CuOOCu, the Al ions are placed opposite each other in the eight-membered ring. The simulated H_2_-TPR profiles show peaks shifted by 10 and 90 °C to higher temperatures for the alternative Al distribution for Z_2_Cu and Z_2_CuOOCu, respectively. Thus, the sensitivity of the TPR profiles on the Al distribution are in these cases not substantial (see the Supporting Information for reaction landscapes and simulated H_2_-TPR profiles).

Some H_2_-TPR studies have reported an H_2_/Cu ratio close to or slightly below 0.5, indicating that one H atom reacts per Cu atom.^2,12^ The effective H_2_/Cu ratio for the reactions over ZCuOH and Z_2_CuOOCu is 1, whereas it is 0.5 over Z_2_CuOCu, Z_2_Cu, and Z_2_CuHOOHCu. This suggests that Z_2_CuOCu, paired Z_2_Cu, and Z_2_CuHOOHCu dominate the experimental samples. It should be stressed that the analysis of the H_2_/Cu is based on the assumption that all Cu ions in the sample are reduced, which may not be valid for samples with a distribution of Cu species.

## Conclusions

We have used density functional theory to study the reaction of H_2_ with selected Cu sites in Cu-CHA. The energy landscapes have been used as input to microkinetic modeling of H_2_-TPR profiles. In addition, we have measured H_2_-TPR profiles from samples that have been selectively prepared to contain dominant Cu species. The comparison between simulated and measured H_2_-TPR profiles allows us to identify and link the peaks in the H_2_-TPR profiles with certain Cu species. In the simulations we find that H_2_ consumption over Cu species that in the +2 oxidation states (Z_2_CuOCu, ZCuOH, Z_2_Cu, [Cu_2_(NH_3_)_4_O_2_]^2+^, Z_2_CuHOOHCu, and Z_2_CuOOCu) From typical H_2_-TPR profiles reported in the literature, we assign the low-temperature peak at 220 °C to ZCuOH, the medium-temperature peak at 360 °C to Z_2_CuHOOHCu and paired Z_2_Cu species, and the high-temperature peak at 500 °C to paired Z_2_Cu species and Z_2_CuOOCu. Our study demonstrates the possibility to use DFT in combination with microkinetic modeling to interpret H_2_-TPR measurements, which enables linking the presence of certain Cu species with catalyst function.

## Supporting Information Available

Additional settings for the microkinetic model and constrained molecular dynamic simulations together with energy landscapes and measured NH_3_ desorption profiles (PDF)
