# Supplementary material for: Interpretation of H2-TPR from Cu-CHA Using First-Principles Calculations
Source: J Phys Chem C Nanomater Interfaces. 2024 Mar 8;128(11):4525–34. doi: 10.1021/acs.jpcc.3c07998 (PMC10962680; doi:10.1021/acs.jpcc.3c07998)
Supplement: Supplementary file 1 — jp3c07998_si_001.pdf [file jp3c07998_si_001.pdf]

Supporting Information: On the Interpretation of H_2-TPR from Cu-CHA using First-Principles Calculations

Joachim D. Bjerregaard,*,† Joonsoo Han,‡ Derek Creaser,‡ Louise Olsson,‡ and Henrik Grönbeck*,†

†*Department of Physics and Competence Centre for Catalysis, Chalmers University of Technology, SE-412 96 Göteborg, Sweden*

‡*Chemical Engineering and Competence Centre for Catalysis, Chalmers University of Technology, Gothenburg, SE 412 96 Göteborg, Sweden*

E-mail: joabje@chalmers.se; ghj@chalmers.se

Constrained Molecular Dynamic Simulations

For the reaction of H_2 with $[Cu_2(NH_3)_4O_2]^{2+}$, it was not possible to locate the transition state using CI-NEB. Instead, ab initio molecular dynamics using the slow growth method as implemented in VASP, is used to probe the reaction barrier. The temperature is set to 300 K and is controlled using a Nosé-Hoover thermostat in the NVT ensemble. The mass of hydrogen is set to 3, and the time step is 1 fs. The collective variable is the sum of distance r1 and r2, illustrated in Figure S1. The step size was set to -0.0004 Å giving a total simulation time of $\sim$ 10 ps.

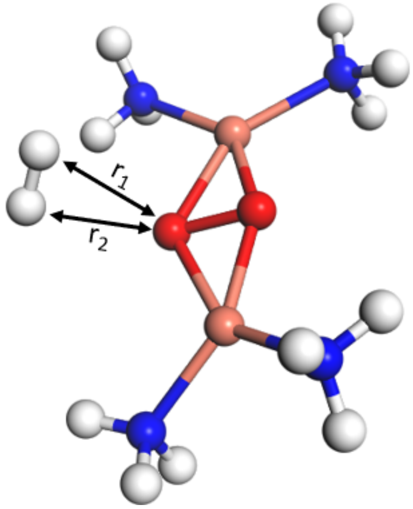

Figure S1: Structure of $[Cu_2(NH_3)_4O_2]^{2+}$ illustrating the collective variable chosen for the simulation. Atomic color codes: H(white), N(blue), O(red), S(yellow) and Cu(Bronze).

Adsorption of H_2 on $[Cu_2(NH_3)_4O(H_2O)]^{2+}$

The reaction of H_2 with $[Cu_2(NH_3)_4O_2]^{2+}$ forms $[Cu_2(NH_3)_4O(H_2O)]^{2+}$. $[Cu_2(NH_3)_4O(H_2O)]^{2+}$ can further react with a second H_2 molecule and this reaction is shown in Figure S2. The barrier is 1.0 eV and the reaction is exothermic by 2.9 eV. During the start of the simulation, a single NH_3 molecule is desorbed from the complex and diffuses into an adjacent cage and is therefore not shown in the structures. The H_2 molecule is dissociated over the Cu ion forming an additional H_2O and reducing both Cu ions to oxidation state +1.

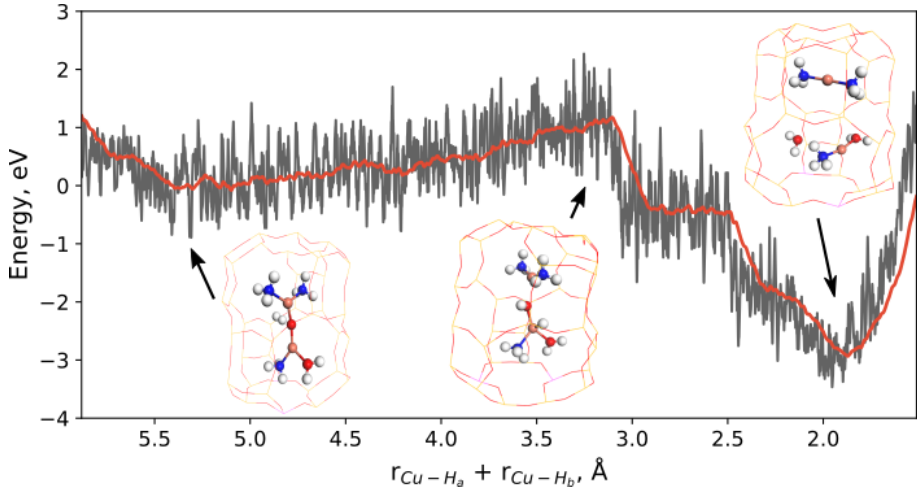

Figure S2: Constrained AIMD simulations of the energy profile for the reaction of H_2 with $[Cu_2(NH_3)_4O(H_2O)]^{2+}$. The gray line is the energy and the red line is the rolling average of the energy. Atomic color codes as in Figure S1.

H_2 Adsorption on ZCu

H_2 can adsorb on ZCu with a low adsorption energy of -0.28 eV. The adsorbed state is shown in Figure S3. A possible reaction could be the formation of a Brønsted acid site and a Cu-H complex. That is similar to the reaction pathway for H_2 over Z_2Cu (See Figure 4, Structure 5). However, for the case of ZCu, the Cu ion will not have any associated Al.

$$\mathrm{ZCu} + \mathrm{H_2} \longrightarrow \mathrm{ZH} + \mathrm{CuH} \tag{R1}$$

Several configurations of ZH + CuH were optimized, however, no minimum was located.

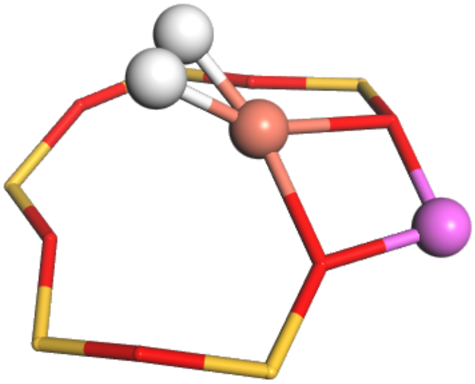

Figure S3: Optimized structure for the adsorption of H_2 onto ZCu. Atomic color codes as in Figure S1.

Reaction of ZCuH with ZCuOH

The product of the reaction between ZCuOH and H_2 is ZCuH and H_2O. ZCuH could potentially reduce a second ZCuOH species and the energy landscape for this reaction is shown in Figure S4. The OH-group in ZCuOH can bind to ZCuH forming Z_2CuOHCuH, which has a barrier of 0.09 eV and is exothermic by -0.2 eV. The next step is a slight movement in the Cu ion and is endothermic by 0.07 eV. The OH-group can subsequently be transferred to ZCuH forming H_2O and reducing both Cu ions to oxidation state +1 (ZCu + ZCuH_2O). This process is exothermic by -1.83 eV.

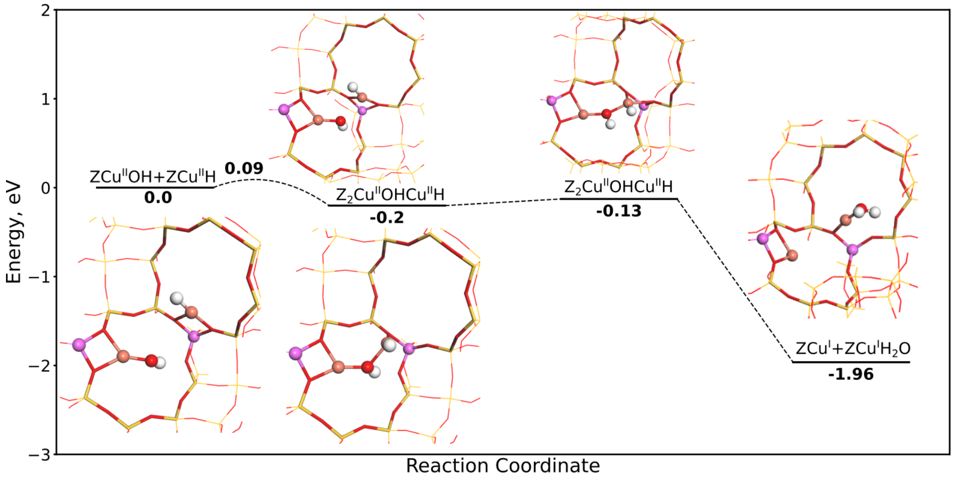

Figure S4: Energy landscape for the reaction of ZCuH with ZCuOH. Atomic color codes as in Figure S1.

Effect of Al-Distributions

H_2 dissociation over paired Z_2Cu and $Z_2CuOOCu$ is calculated for alternative Al-distributions by considering the most important steps. For Z_2Cu, the Al ions are placed in an Al-O-Si-O-Al configuration and the reaction landscape is shown in Figure S5(a). H_2 is dissociated over Z_2Cu, forming $Z_2Cu + ZHZCuH$. This step has a barrier of 0.88 eV and is endothermic by 0.65 eV. The following step is the transfer of the hydrogen to form a Brønsted acid site, which reduced the two Cu ions ($Z_2Cu_2 + Z_2H_2$). The hydrogen transfer has a barrier of 0.43 eV and is exothermic by 1.87 eV. The reaction landscape should be compared with H_2 over paired Z_2Cu in Figure 4 (structure 6). The barrier for the first step ($Z_2Cu + Z_2Cu + H_2 \rightarrow Z_2Cu + ZHZCuH$) is increased by 0.08 eV for the Al configuration in Figure S5(a) and is 0.09 eV more endothermic. The barrier for the transfer of the hydrogen is 0.89 eV lower and is 1.08 eV more exothermic.

For $Z_2CuOOCu$ the dissociation of H_2 is shown in Figure S5(b), where the Al ions are placed opposite of each other. The reaction is endothermic by 1.12 eV. This should be compared to Figure 4 (structure 8). The reaction shown in S5(b) is 0.12 eV more endothermic.

Simulated H_2-TPR profiles of the alternative Al-configurations for Z_2Cu and $Z_2CuOOCu$ are shown in Figure S5(c) (dashed lines), compared to the simulated H_2-TPR profiles reported in the paper (solid lines). For Z_2Cu and $Z_2CuOOCu$, the reduction temperature is increased by 10 °C and 90 °C, respectively, with respect to the Al-configurations in the main text.

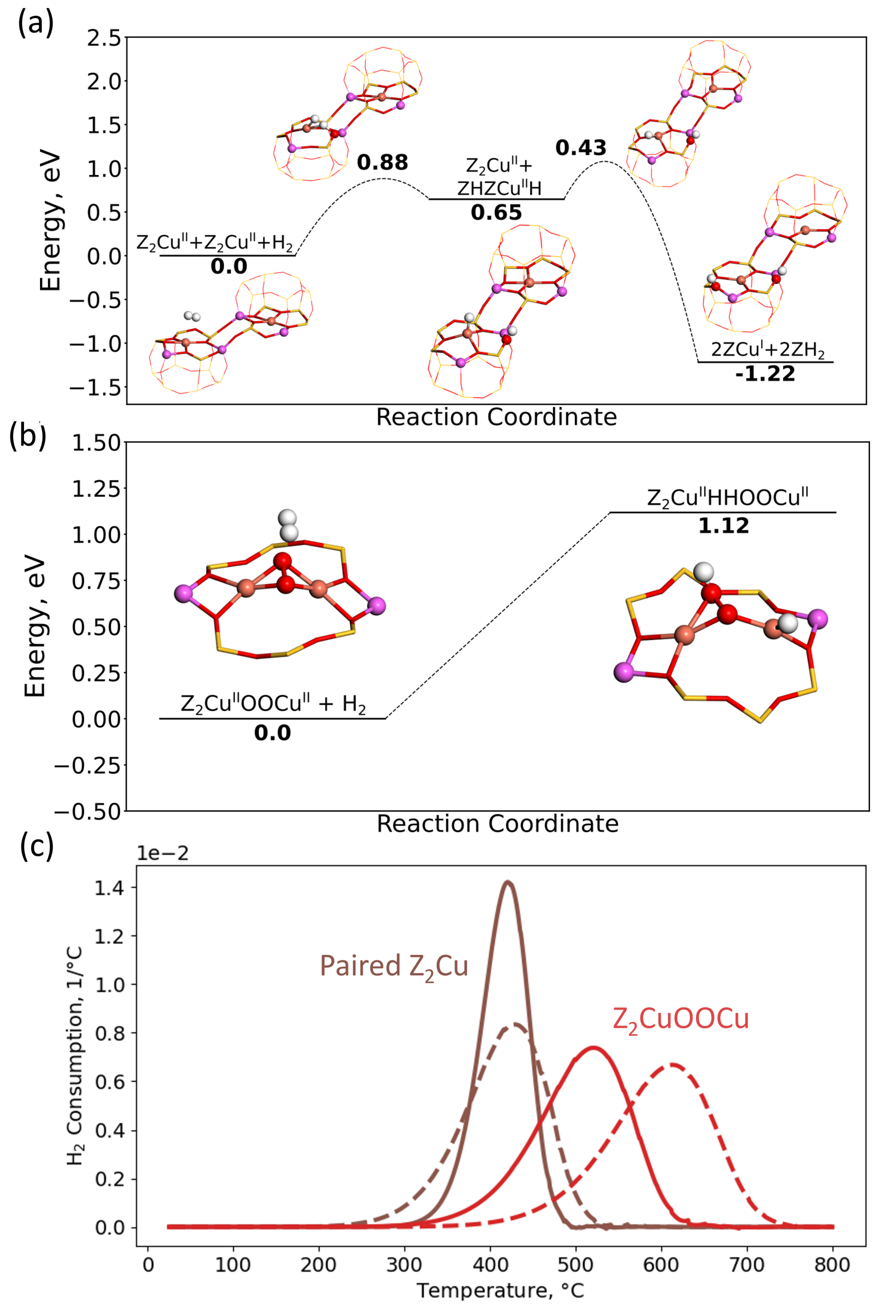

Figure S5: Reaction landscape for the dissociation of H_2 over (a) Z_2Cu and (b) $Z_2CuOOCu$, with alternative Al-configurations. (c) H_2-TPR simulated profiles of the alternative Al configuration (dashed lines) and the simulated H_2-TPR profiles in the manuscript (solid lines). Atomic color codes as in Figure S1.

Formation of Z_2CuHOOHCu from two ZCuOH Species

A pair of ZCuOH species can form a Z_2CuHOOHCu complex. The reaction landscape is shown in Figure S6. First the OH-group binds to both Cu ions forming Z_2CuOHCuOH. This step has a low barrier of 0.09 eV and is exothermic by -0.11 eV. Also the second OH-group binds to both Cu ions forming Z_2CuHOOHCu. This process has a barrier of 0.1 eV and is exothermic by -1.13 eV.

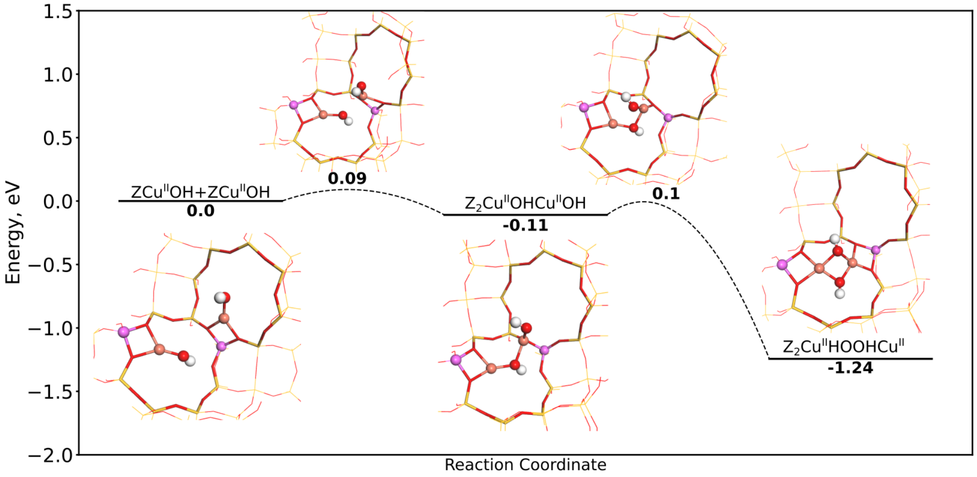

Figure S6: Energy landscape for the formation Z_2CuHOOHCu from two ZCuOH complexes. Atomic color codes as in Figure S1.

Elementary Steps and Kinetic Parameters

The considered reaction steps and associated kinetic parameters are reported in Table S1.

Table S1: Elementary steps included in the microkinetic model. Prefactors and barriers are given for the forward (A_f, ΔE_f) and reverse (A_f, ΔE_f) reaction. The parameters are evaluated at 200 °C for 2000 ppm H_2 in Ar.

Species	No.	Elementary step	A_f (s^{-1})	A_r (s^{-1})	ΔE_f (eV)	ΔE_r (eV)
**$[Cu_2(NH_3)_4O_2]^{2+}$**	1	$[Cu_2(NH_3)_4O_2]^{2+} + H_2 \rightleftharpoons [Cu_2(NH_3)_4O(H_2O)]^{2+}$	$8.70 \cdot 10^3$	$7.26 \cdot 10^5$	0.92	3.4
**Isolated Z_2Cu**	1	$Z_2Cu + H_2 \rightleftharpoons ZCuH + ZH$	$8.70 \cdot 10^3$	$2.5 \cdot 10^{12}$	0.51	0.19
**Paired Z_2Cu**	1	$Z_2Cu + Z_2Cu + H_2 \rightleftharpoons Z_2Cu + ZHZCuH$	$8.70 \cdot 10^3$	$3.47 \cdot 10^8$	0.8	0.24
	2	$Z_2Cu + ZHZCuH \rightleftharpoons 2\,ZCu + 2\,ZH_2$	$2.08 \cdot 10^{16}$	$8.05 \cdot 10^{14}$	1.32	2.11
	3	$2\,ZCu + 2\,ZH_2 \rightleftharpoons 2\,ZCu + 2\,ZH_2$	$2.28 \cdot 10^{14}$	$3.43 \cdot 10^{14}$	0.62	1.03
	4	$2\,ZCu + 2\,ZH_2 \rightleftharpoons 2\,ZCu + 2\,ZH_2$	$6.19 \cdot 10^{13}$	$6.86 \cdot 10^{13}$	0	0.11
	5	$2\,ZCu + 2\,ZH_2 \rightleftharpoons 2\,ZCu + 2\,ZH_2$	$4.77 \cdot 10^{14}$	$4.27 \cdot 10^{14}$	0.58	0.66
**ZCuOH**	1	$ZCuOH + H_2 \rightleftharpoons ZCuH{-}H_2O$	$8.70 \cdot 10^3$	$6.75 \cdot 10^7$	0.52	0.77
	2	$ZCuH{-}H_2O \longrightarrow ZCuH + H_2O$	10^{12}	-	0.95	-
**$Z_2CuOOCu$**	1	$Z_2CuOOCu + H_2 \rightleftharpoons Z_2CuHHOOCu$	$8.70 \cdot 10^3$	$1.13 \cdot 10^8$	1.0	0.0
	2	$Z_2CuHHOOCu \rightleftharpoons Z_2CuHOCuOH$	$6.19 \cdot 10^{13}$	$3.54 \cdot 10^{13}$	0.0	3.5
	3	$Z_2CuHOCuOH \rightleftharpoons Z_2CuHOOHCu$	$3.26 \cdot 10^{13}$	$1.42 \cdot 10^{16}$	0.12	1.28
**$Z_2CuHOOHCu$**	1	$Z_2CuHOOHCu \rightleftharpoons Z_2CuOHCuOH$	$8.20 \cdot 10^{14}$	$6.19 \cdot 10^{13}$	1.5	0
	2	$Z_2CuOHCuOH \rightleftharpoons ZZ_2CuOCuH_2O$	$7.10 \cdot 10^{14}$	$6.19 \cdot 10^{13}$	0.05	0.08
	3	$ZZ_2CuOCuH_2O \longrightarrow Z_2CuOCu + H_2O$	10^{12}	-	0.4	-
	4	$Z_2CuOCu + H_2 \rightleftharpoons Z_2CuOHCuH$	$8.70 \cdot 10^3$	$8.30 \cdot 10^8$	0.45	0.97
	5	$Z_2CuOHCuH \rightleftharpoons Z_2CuOCuH_2O$	$6.19 \cdot 10^{13}$	$1.58 \cdot 10^{15}$	0	1.75
	6	$Z_2CuOCuH_2O \longrightarrow Z_2Cu_2 + H_2O$	10^{12}	-	1.85	-
**Z_2CuOCu**	1	$Z_2CuOCu + H_2 \rightleftharpoons Z_2CuOHCuH$	$8.70 \cdot 10^3$	$8.30 \cdot 10^8$	0.45	0.97
	2	$Z_2CuOHCuH \rightleftharpoons Z_2CuOCuH_2O$	$6.19 \cdot 10^{13}$	$1.58 \cdot 10^{15}$	0	1.75
	3	$Z_2CuOCuH_2O \longrightarrow Z_2Cu_2 + H_2O$	10^{12}	-	1.85	-

Release of NH_3 during H_2-TPR

Experiments (a) and (b) in Figure 6 of the main text are pretreated to have $[Cu(NH_3)_2]^+$ and $[Cu_2(NH_3)_4O_2]^{2+}$ dominating, respectively. During the H_2-TPR measurements, NH_3 release is observed as shown in Figure S7. Desorption of NH_3 begins at 300 °C indicating that $[Cu(NH_3)_2]^+$ and $[Cu_2(NH_3)_4O_2]^{2+}$ starts to decompose at this temperature.

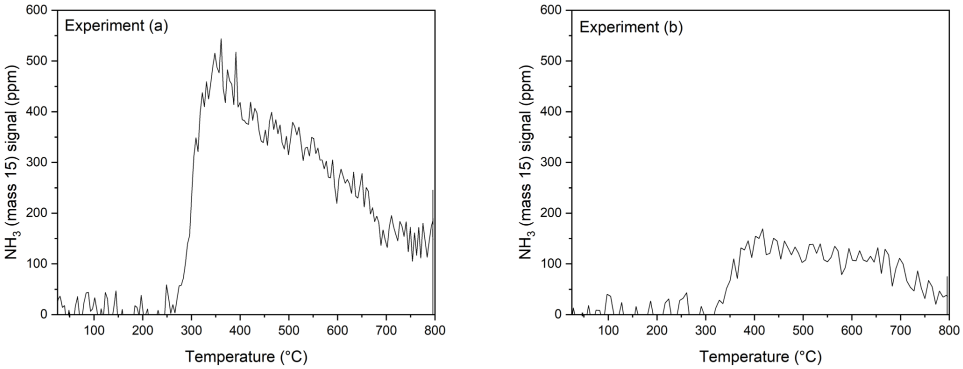

Figure S7: Measured NH_3 desorption profile during H_2-TPR for experiments (a) and (b).
